# Perceived discrimination and relative deprivation in Chinese migrant adolescents: the mediating effect of locus of control and moderating effect of duration since migration

**DOI:** 10.1186/s13034-021-00436-9

**Published:** 2022-01-08

**Authors:** Meng Xiong, Wendy Johnson

**Affiliations:** 1 Department of Psychology, Yangtze University, Jingzhou, 434023 Hubei China; 2 Department of Psychology, University of Edinburgh, Edinburgh, UK

**Keywords:** Perceived discrimination, Relative deprivation, Locus of control, Migrant adolescents, Duration since migration

## Abstract

**Background:**

Associations between perceived discrimination and relative deprivation have been observed among both general and migrant populations. However, it is unclear how, and under what conditions, perceived discrimination relates to relative deprivation, a subjective cognition and affective experience in which individuals or groups perceive themselves as disadvantaged, compared to their peers. Therefore, this study aimed to construct a moderated mediation model to examine the roles of locus of control and duration since migration in the relationship between perceived discrimination and relative deprivation among Chinese rural-to-urban migrant adolescents.

**Methods:**

We conducted a cross-sectional study using a convenience sampling method in three coastal cities in southeast China. We recruited 625 Chinese rural-to-urban migrant adolescents, who completed a battery of questionnaires assessing perceived discrimination, relative deprivation, locus of control, and demographic variables. Regression-based statistical mediation and moderation were conducted using the PROCESS macro for SPSS.

**Results:**

After controlling for sex and age, perceived discrimination was positively associated with migrant adolescents’ relative deprivation, and external locus of control partially mediated this connection. Furthermore, the mediating effect was moderated by the duration of the migration. In relatively recently migrated adolescents, perceived discrimination was significantly related to relative deprivation through a greater external locus of control; however, this indirect association was not significant for adolescents with long-term migratory duration.

**Conclusion:**

The results of our analysis expand our understanding of the link between perceived discrimination and relative deprivation. Moreover, these findings may provide practical guidance for interventions among Chinese rural-to-urban migrant adolescents to raise their social status and improve their mental health by addressing the macro-social psychological causes of relative deprivation.

## Introduction

Rural-to-urban migration for obtaining job opportunities and higher incomes has become a global phenomenon [1]. During the past four decades, China has experienced the largest scale of rural-to-urban migration in human history [2]. Many migrant workers leave their children in their rural hometowns in the care of their grandparents, while others bring them along to their destination cities [3]. Due to differences in culture, values, lifestyles, and socioeconomic status (SES) between urban and rural regions, migrant children and adolescents often encounter social exclusion, prejudice, and discrimination as they adjust to city life [2, 4]. For example, a recent survey reports that approximately 20% of migrant students had experienced origin-based discrimination in urban schools [5]. Higher levels of perceived discrimination are linked to more physical and mental health problems [6–8], and may jeopardize general social stability and harmony [9]. The concept of *relative deprivation* has been useful in understanding migrant populations’ reactions to perceived discrimination and social exclusion [10, 11]. The concept refers to an individual’s anger and/or discontent stemming from the perception that they—or their group—are being deprived of deserved outcomes, compared with referent individuals or groups [12, 13]. Empirical evidence also indicates that perceived discrimination may lead to individual and group-relative deprivation [11, 14, 15]. However, little is known about how perceived discrimination affects migrant adolescents’ relative deprivation and the conditions under which the influence is stronger or weaker.

### Perceived discrimination and relative deprivation

Overtly discriminative behavior tends to be socially repugnant, which makes it rather rare. However, discrimination is often manifested in subtle ways in daily life; this makes it difficult to measure discrimination objectively [7]. Therefore, researchers shifted from assessing discrimination that is experienced objectively to discrimination that is perceived subjectively. *Perceived discrimination* is the subjective perception of prejudicial and/or distinguishing treatment of an individual based on membership in socially recognized groups or specific personal characteristics, including race, gender, disability, ethnic origin, immigration status, social class, or other manifestations of cultural background [16]. Individuals’ pre-existing psychological characteristics are closely related to their perception, and thus, their assessment [8, 17, 18].

Social comparison theory suggests that individuals evaluate their situations in comparison with similar others [19, 20]. Chinese adolescents who migrate from rural to urban areas with their parents may face difficulties that their urban-native counterparts have not experienced. For example, migrant adolescents with non-urban *hukou*—a household registration that officially identifies a person as a permanent resident of an area and includes information such as name, sex, parents, native place, and date of birth—are not allowed to enroll in the city schools and/or are required to take higher-level school entrance examinations at their *hukou* localities [8]. As a result, these adolescents may feel that the world is an unjust place where people cannot get what they deserve (cognitive components of relative deprivation [14], and they may be frustrated and dissatisfied with their situation (affective components of relative deprivation [21]. Meanwhile, according to the relative deprivation theory [22–24], the development of relative deprivation involves three related elements: first, individuals become aware of the differences between themselves and others whom they consider relevant to them by making social comparisons; second, they evaluate whether the perceived differences seem justified (cognitive component of relative deprivation; and third, upon concluding that they are not justified, they may feel anger or resentment (emotional component of relative deprivation; [25, 26].

Moreover, migrant adolescents may frequently encounter discrimination and stigmatization, based on differences in how they dress and speak, and have difficulty interacting with other students, due to unfamiliarity with common local games and other social forms of entertainment [27]. Hence, migrant adolescents may feel deprived and treated unjustly, compared with their urban or rural peers [13, 28], this perception of discrimination may give rise to feelings of relative deprivation [11]. Furthermore, several empirical studies demonstrate that perceived discrimination influences relative deprivation in disadvantaged groups (e.g., migrant populations [10, 11, 14, 15]. Other existing research also indicates that family environment (e.g., family SES) and school environment (e.g., perceived classroom climate) can play significant roles in migrant adolescents’ relative deprivation [29]. Accordingly, we propose that the social environment (e.g., discrimination) would also influence relative deprivation among migrant adolescents. Therefore, our first hypothesis is that perceived discrimination is positively associated with relative deprivation among Chinese migrant adolescents (H1).

### Mediating role of locus of control

Although considerable research indicates the influence of perceived discrimination on relative deprivation, few studies have explored the intermediary processes. The *locus of control* from social learning theory [30] reflects people’s beliefs about the degrees to which they can control events and outcomes in their lives [31, 32]. The locus of control is conceptualized as being relatively internal or external [33]. Individuals with a strong internal locus of control believe they can do much to control their fates and that their efforts do much to affect their personal achievements and outcomes [31, 34, 35]. Individuals with a relatively external locus of control feel that their lives are largely determined by outside factors (e.g., chance, luck, powerful others, or social structures [30, 36, 37]. Those with a more internal locus of control tend to believe they can manage negative or threatening events (e.g., discrimination; [38] to mitigate negative consequences, while those with a more external locus of control are more likely to experience anxiety and withdrawal in such situations; moreover, withdrawal may aggravate negative consequences [39]. Previous studies indicate that experiencing stressful events (e.g., discrimination) can shift the locus of control externally [35, 40]. Furthermore, a growing body of research observes that perceived discrimination is significantly associated with an external locus of control among disadvantaged groups [33, 38, 41, 42]. Hence, the current study suggests that perceived discrimination is positively related to an external locus of control among migrant adolescents.

Furthermore, high levels of an external locus of control have been associated with a series of mental health problems, including depression [43, 44], anxiety [33], and psychotic experiences [45]. In general, members of migrant populations tend to feel less control over their situations—especially when they are newly arrived—because of their relative unfamiliarity with their new environment [46]. They may compare their situations with those of their new urban-native counterparts and often feel that they have fewer material resources or less than they should be entitled to receive [47], if only because rural Chinese areas tend to have lower wage levels and fewer and/or poorer socially provided resources [48]. Thus, members of disadvantaged groups with an external locus of control tend to experience a greater sense of relative deprivation than members of advantaged groups [49, 50].

Empirical studies also indicate that higher levels of external locus of control are associated with higher levels of relative deprivation among disadvantaged groups [21, 46, 51]. According to Bronfenbrenner’s [52] ecological systems theory, the interaction between individuals and their surrounding environments influences their psychosocial development. The theory regards individual characteristics as crucial to understanding and explaining people’s developmental processes; thus, perceived discrimination (an environmental factor) may affect one’s experience of relative deprivation via individual variables (i.e., locus of control; [51, 53]. Previous research also indicates that locus of control plays a mediating role between stressful events and psychopathological symptoms in children and adolescents [53–56]. Therefore, this study investigates whether locus of control mediates the association between perceived discrimination and relative deprivation among Chinese migrant adolescents (H2).

### Moderating role of the duration since migration

Adolescents and their families, schools, and communities may develop, showing both systematic changes and seemingly spontaneous changes over time [57, 58]. These changes are inevitably rooted in contexts that vary from highly specific to extremely general. *Duration since migration*, the amount of time since the family moved to the new area, provides some context for the situations contributing to changes and problems related to adapting to a new environment; problems related to the change include perceived discrimination and relative deprivation. However, this context may not be entirely concrete, as the move may have appeared to have been a spontaneous change of every aspect of their rural environments and their family characteristics, from children’s perspective. For instance, for parents who planned the move well in advance, the duration since migration began in those planning stages; however, if the parents suddenly announce the move is coming without having previously discussed it and the reasons behind it with the children, the children’s “duration since migration” is shorter and the change—which, for adolescents, will have impacted every aspect of new lives as migrant adolescents (e.g., biological, psychological, cultural, and socio-ecological levels of organization)—is more abrupt [57].

One of the contextual changes that migrant adolescents often experience is finding themselves with relatively lower social status in their new urban environments, than in their previous rural settings [59]. As a result, their perceptions of the external environment and internal psychological characteristics may change while residing in destination cities [60]. To a certain extent, such changes are systematic over resident time; thus, the duration since migration may be an important demographic variable when exploring the relationships between environmental and individual factors and the sense of relative deprivation in migrant populations [29, 60]. Some researchers point out that migrants may integrate into their destinations over time [61–63]. In particular, one empirical study indicates that migrant adolescents’ sense of relative deprivation decreases with the prolongation of their migratory duration in cities [15].

Over time, with a longer duration since migrating to destination cities, migrant adolescents gradually integrate into urban lives and develop new coping skills. As a result, their ability to cope with external and internal risk factors improvise constantly, and their perception of differences from their urban counterparts consequently declines [64]. Hence, the duration since migration may be an important variable to control for in assessing the impacts of stressful events (e.g., discrimination) and individual factors (e.g., external locus of control) on migrant adolescents’ sense of relative deprivation [15]. According to the developmental systems theory [57, 65], which emphasizes the shared contributions of genes, environment, and evolution on development, the emergence of migrant adolescents’ relative deprivation is a dynamic process; this process involves interactions among environmental variables, psychological factors, and duration since migration. Increasingly, studies have also indicated that duration since migration is an important time factor moderating the relationships between family SES and relative deprivation [29] and between perceived discrimination and self-esteem among migrant adolescents [60]. Therefore, we anticipate that duration since migration would moderate the relationship between perceived discrimination and relative deprivation (H3a) and between external locus of control and relative deprivation (H3b) among Chinese migrant adolescents.

### The current study

Considering the above background, this study examined (i) the impact of perceived discrimination on relative deprivation among Chinese migrant adolescents; (ii) whether the locus of control mediated this relationship; and (iii) whether duration since migration moderated the relationship between perceived discrimination and relative deprivation and between external locus of control and relative deprivation. To our knowledge, a growing body of literature on relative deprivation considers only the influence of relative deprivation on psychological and behavioral outcomes among adults [13, 66, 67], with little being known about the factors influencing relative deprivation itself and about the influence of these factors among adolescents.

**Fig. 1:**
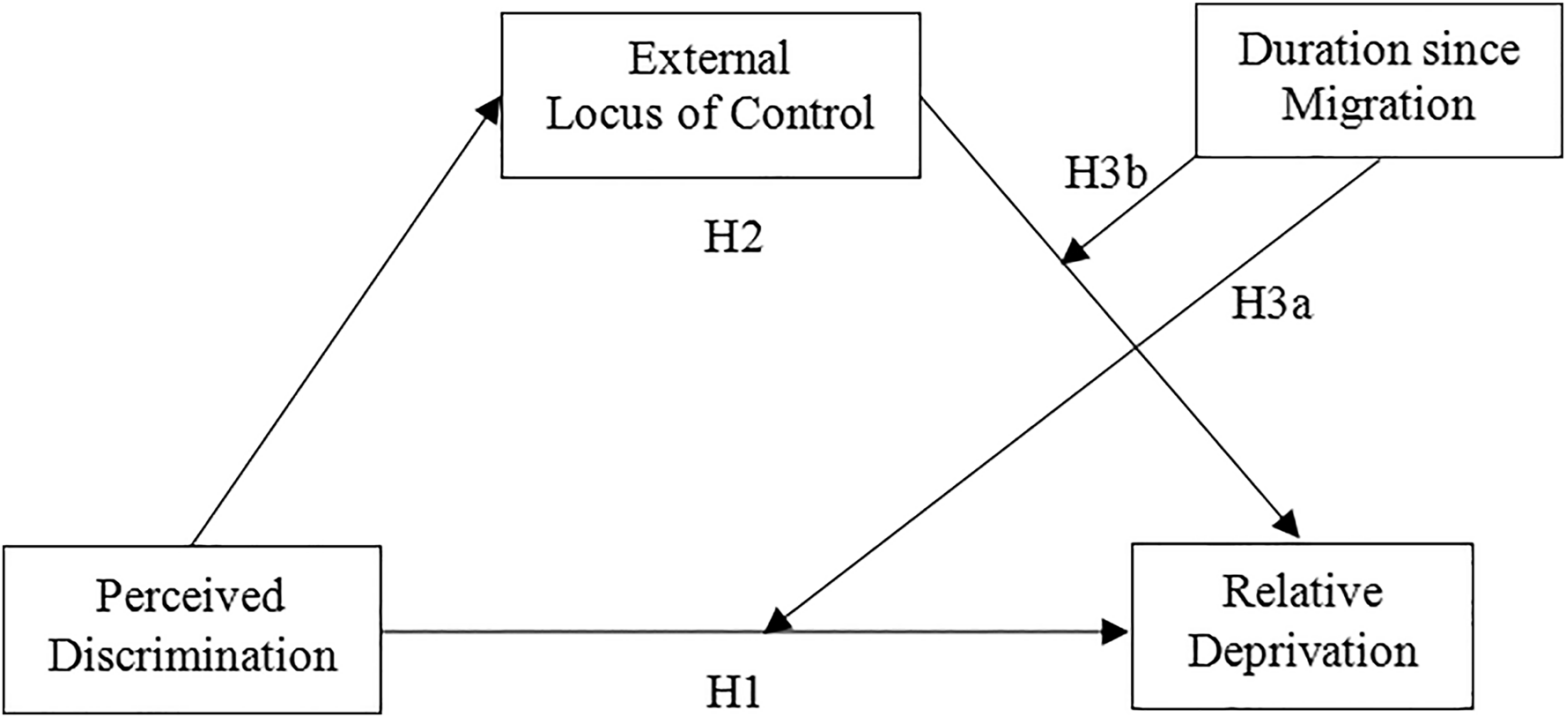
Moderated mediation model of the current study

We focused on a sample of migrant Chinese adolescents because existing studies on relative deprivation have overlooked this large and potentially vulnerable group. Migrant adolescents not only have to complete age-typical developmental tasks (e.g., finding ways to take on adult roles that suit their still-emerging adult identities; see [68], they often must also overcome challenging circumstances (e.g., adjusting to unfamiliar city life, encountering apparent/recessive discrimination or prejudice [69]. These challenging circumstances may affect feelings of relative deprivation, which impair the ability to attain age-typical developmental milestones and adapt to their new environments [13, 26]. In summary, we developed a moderated mediation model to investigate our stated hypotheses. Figure 1 shows the proposed model.

## Methods

### Participants

We recruited 720 migrant adolescents from three primary schools and three junior middle schools in Fuzhou, Xiamen, and Quanzhou—three coastal cities in southeast China. From each of the cities, we selected one primary school (fourth to sixth graders who possessed sufficient literacy skills to complete our questionnaires) and one junior middle school (seventh to ninth graders). Based on previous research [70], our inclusion criteria were as follows: (i) birthplace in a rural environment without urban *hukou*; (ii) living only in rural areas before moving to their current residences; and (iii) accompanying their parents to their current residences, with the intention of remaining for more than six months. Using these, we validated 625 participants, which accounted for 87% of the total recruited sample.

The participants had a mean age of 12.8 years (*SD* = 1.8, ranging from 9 to 17 years). Of these, 321 (51%) were male, and 304 (49%) were female, while 226 (36%) were from primary schools and 399 (64%) were from junior high schools. There were 93 (15%), 382 (61%), and 150 (24%) participants whose duration since migration was fewer than five years, from five to ten years, and more than ten years, respectively. All participants’ parents or legal guardians provided written informed consent before the survey. Our study was performed in accordance with the Declaration of Helsinki but was approved by the Ethics Committee for Academic Research at the corresponding author’s institution.

### Measures

#### Perceived discrimination

We assessed migrant adolescents’ perceived discrimination using the Perceived Personal Discrimination Scale developed by Liu and Shen [64]. This scale assesses the degree of discrimination perceived by migrant adolescents when engaging in social interactions with their peers. It comprises 20 items regarding typical discrimination incidents sometimes experienced by migrant adolescents in schools and in their social lives (e.g., *Some local children do not like to play with me; I have been laughed at by local people on some public occasions*). Items are rated on a 5-point Likert scale ranging from 1 (*completely inconsistent*) to 5 (*completely consistent*), with higher scores indicating higher levels of perceived discrimination. This scale has been used in many studies, with good reliability and validity [7, 8]. The data of this study showed that the fit indexes of the scale were good (comparative fit index [CFI] = 0.94, Tucker-Lewis Index [TLI] = 0.94, χ^2^/degrees of freedom [*df*] = 13.26, standardized root mean square residual [SRMR] = 0.067). In this study, the internal consistency of the scale (Cronbach’s α) was 0.85.

#### Relative deprivation

We used the Relative Deprivation Scale for Migrant Adolescents (RDS-MA), developed by Xiong [15], to measure migrant adolescents’ sense of relative deprivation. The measure assesses relative deprivation on four dimensions: individual cognitive relative deprivation, individual emotional relative deprivation, group-based cognitive relative deprivation, and group-based emotional relative deprivation. This scale refers to five aspects of migrant adolescents’ current situations: family economic status, housing conditions, residential stability, development of personal strengths, and parental involvement in education (e.g., *How do you compare your family*’*s economic status with that of your urban counterparts*? *How satisfied are you with this situation*?). The scale consists of 20 items rated on a 7-point Likert scale. The items of the cognitive dimension range from 1 (*very good*) to 7 (*very bad*), while the items of the emotional dimension ranged from 1 (*very satisfied*) to 7 (*extremely unsatisfied*, higher scores indicate higher levels of relative deprivation. The RDS-MA has shown excellent internal consistency (*α* = 0.92), strong test–retest reliability over four weeks (*r* = 0.80; [15], and good construct validity (CFI = 0.93, TLI = 0.92, χ^2^/*df* = 11.14, SRMR = 0.073). In this study, the internal consistency of the scale was 0.93.

#### Locus of control

We measured migrant adolescents’ locus of control using the Internal–External Locus of Control Scale [30], which comprises 23 items. Each item consists of two statements, internal locus of control (self-blame) or external locus of control (fate-blame) over outcomes of life events, from which respondents chose 1 (e.g., “*I often find out what’s going to happen before it actually happens”* vs. “*For me, it’s better to believe in fate than to struggle”*). The total scale score ranges from 0 (*extremely internal locus of control*) to 23 (*extremely external locus of control*). The scale has been widely used in adolescent groups with good reliability and validity [71, 72]. Structural validity (CFI = 0.94, TLI = 0.92, χ^2^/*df* = 1.95, SRMR = 0.046) was in line with the standards of psychometrics. In this study, the internal consistency of the scale was 0.76.

### Data analysis

Statistical analyses were performed using IBM SPSS version 22.0. We first calculated the descriptive statistics and correlations for the key variables. We then used the SPSS PROCESS macro, developed by Hayes ([73], http://www.afhayes.com), to assess the mediating role of locus of control and the moderating effect of duration since migration in the relationships between perceived discrimination and relative deprivation, as well as in the relationships between locus of control and relative deprivation. The macro has been widely used in previous studies to evaluate moderated mediation or mediated moderation models with the bias-corrected percentile bootstrap method [74–76]. Additionally, because previous studies found sex and age differences in migrant adolescents’ perceived discrimination [7, 77] and relative deprivation [26, 78], we included sex and age as covariates in all our analyses. Furthermore, the potential common method bias effect was examined using Harman’s single factor test for all the research items. The results revealed 17 distinct factors with eigenvalues greater than one; of these, the largest factor accounted for 20.07% of the total variance, which is below the threshold level of 40% [79]. Therefore, common method deviation was not obvious in the present study.

## Results

### Preliminary analyses

The means, standard deviations, and correlations are presented in Table 1. Perceived discrimination was significantly correlated with the relative deprivation (*r* = 0.22, *p* < 0.001) and locus of control (*r* = 0.32, *p* < 0.001) of migrant adolescents. Locus of control was significantly related to relative deprivation (*r* = 0.23, *p* < 0.001), while duration since migration was significantly associated with perceived discrimination (*r* = -0.17, *p* < 0.001), locus of control (*r* = -0.12, *p* < 0.01), and relative deprivation (*r* = -0.16, *p* < 0.001).

**Table 1:** Descriptive statistics and correlations among key variables

Variables	*M ean*	*SD*	1	2	3	4	5	6
1.Sex	.51	.50	–					
2.Age	12.75	1.79	.01	–				
3.Perceived discrimination	1.81	.79	.13^**^	− .11^**^	–			
4.Locus of control	7.94	3.58	.08	.04	.32^***^	–		
5.Duration since migration	7.70	3.65	-.04	.08^*^	− .17^***^	− .12^**^	–	
6.Relative deprivation	3.34	.93	.06	.11^**^	.22^***^	.23^***^	− .16^***^	–

*N* = 625. Sex is a virtual variable: 0 = female students, 1 = male students, the mean represents the proportion of male students; age and duration since migration can be regarded as a continuous variable by using the original scores. *SD* standard deviation

^*^p < .05, ^**^p < .01, ^***^p < .001

### Mediating effect of locus of control

The key variables in our model are continuous variables, and a significant correlation exists between the variables for use in the moderated mediation model test. First, we conducted Model 4 (a single mediation model) of the SPSS PROCESS macro, compiled by Hayes [73], to examine whether an external locus of control mediates the relationship between perceived discrimination and relative deprivation (see Fig. 1). All variables were standardized before the analysis [80]. After controlling for sex and age, we used bootstrap estimates (95% confidence interval [CI]) to evaluate the theoretical model, based on 5,000 bootstrap samples. Perceived discrimination was directly and positively related to migrant adolescents’ relative deprivation (*B* = 0.23, *p* < 0.001, 95% CI [0.14, 0.32]; see Table 2). After including the external locus of control as the mediator, the effect of perceived discrimination on locus of control was significant (*B* = 0.33, *p* < 0.001, 95% CI [0.26, 0.39]), and the association between locus of control and relative deprivation was also significant (*B* = 0.16, *p* < 0.001, 95% CI [0.08, 0.25]). Meanwhile, the direct path from perceived discrimination to relative deprivation was still significant (*B* = 0.18, *p* < 0.001, 95% CI [0.08, 0.27]), indicating that locus of control partially mediated the relationship between perceived discrimination and migrant adolescents’ relative deprivation (indirect effect = 0.05, SE = 0.02, 95% CI [0.03, 0.09]). The indirect association accounted for approximately 22.9% of the total variance. Thus, Hypotheses 1 and 2 were supported.

**Table 2:** Summary of mediation results

Outcome(Y)	Predictors(X)	Model summary					95% CI
		*R*	*R*^*2*^	*F*	*B*	SE	
RD		.26	.07	12.04^***^			
	Sex				.06	.08	[− .09, .22]
	Age				.08^***^	.02	[.04, .12]
	PD				.23^***^	.05	[.14, .32]
LOC		.33	.11	34.30^***^			
	Sex				.07	.08	[− .08, .22]
	Age				.04^*^	.02	[.002, .09]
	PD				.33^***^	.03	[.26, .39]
RD		.31	.09	12.58^***^			
	Sex				.05	.08	[− .10, .20]
	Age				.07^***^	.02	[.03, .11]
	PD				.18^***^	.05	[.09, .27]
	LOC				.16^***^	.04	[.08, .25]

Effect	*B*	Boot SE	Boot LLCI	Boot ULCI
Direct	.18	.05	.09	.27
Indirect	.05	.02	.03	.09

*N* = 625

*PD* perceived discrimination, *RD* relative deprivation, *LOC* locus of control. Bootstrap sample size = 5000. *CI* confidence interval, *LL* low limit, *UL* upper limit

^*^p < .05, ^**^p < .01, ^***^p < .001

### Moderation of duration since migration

We further conducted Model 15 (a moderated mediation model) of PROCESS macro [73] to assess whether duration since migration moderated the direct effect of perceived discrimination on relative deprivation and the indirect effect of external locus of control in the mediation model (see Fig. 1). All variables were standardized. Sex and age were controlled for as covariates. As shown in Table 3, we observed a prominent direct association between locus of control and relative deprivation (*B* = 0.15, *p* < 0.001, 95% CI [0.07, 0.23]), and this association was significantly moderated by the duration since migration (*B* = − 0.08, *p* < 0.05, 95% CI [− 0.16, − 0.01]). Furthermore, although perceived discrimination was positively related to relative deprivation (*B* = 0.18, *p* < 0.001, 95% CI [0.08, 0.27]), this association was not significantly moderated by duration since migration (*B* = 0.06, *p* = 0.14, 95% CI [− 0.03, 0.16]). This indicates that duration since migration moderates the relationship between locus of control and relative deprivation. Thus, Hypothesis 3b was supported.

**Table 3:** Moderated mediation analysis results with locus of control as a mediator

Outcome(Y)	Predictors(X)	Model summary			*B*	SE	95% CI
		*R*	*R*^*2*^	*F*			
RD		.33	.11	8.74^***^			
	Sex				.04	.08	[− .11, .20]
	Age				.07^**^	.02	[.03, .11]
	PD				.18^***^	.05	[.08, .27]
	LOC				.15^***^	.04	[.07, .23]
	DSM				− .11^**^	.04	[− .18, − .03]
	LOC × DSM				− .08^*^	.04	[− .16, − .01]
	PD × DSM				.06	.05	[− .03, .16]

Effect	DSM values	*B*	Boot SE	Boot LLCI	Boot ULCI
	M-1SD (4.05)	.11	.06	-.01	.24
Direct	M (7.70)	.18	.05	.08	.27
	M + 1SD (11.35)	.24	.07	.10	.38
	M-1SD (4.05)	.08	.02	.03	.13
Indirect	M (7.70)	.05	.02	.02	.09
	M + 1SD (11.35)	.02	.02	-.01	.06

*N* = 625. PD, perceived discrimination; RD, relative deprivation; LOC, locus of control; DSM, duration since migration. Bootstrap sample size = 5000

*CI* confidence interval, *LL* low limit, *UL* upper limit

^*^p < .05, ^**^p < .01, ^***^p < .001

**Fig. 2:**
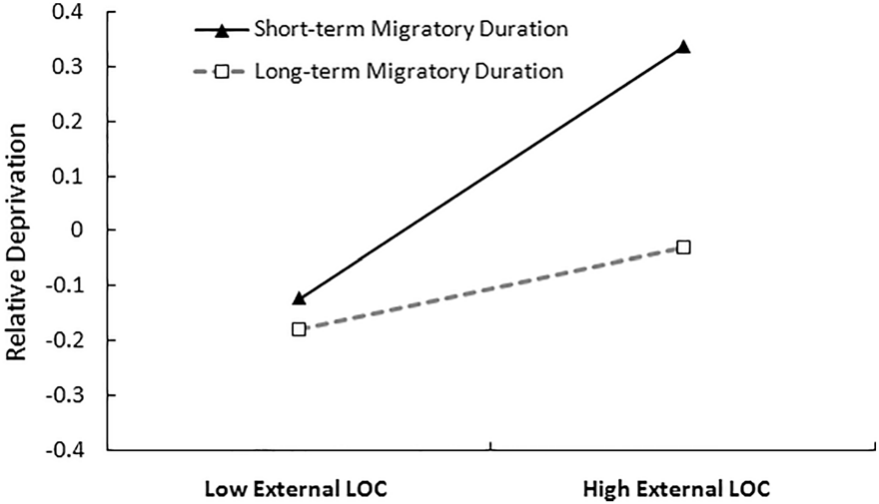
Moderating effect of duration since migration in the relation between locus of control and relative deprivation

**Fig. 3:**
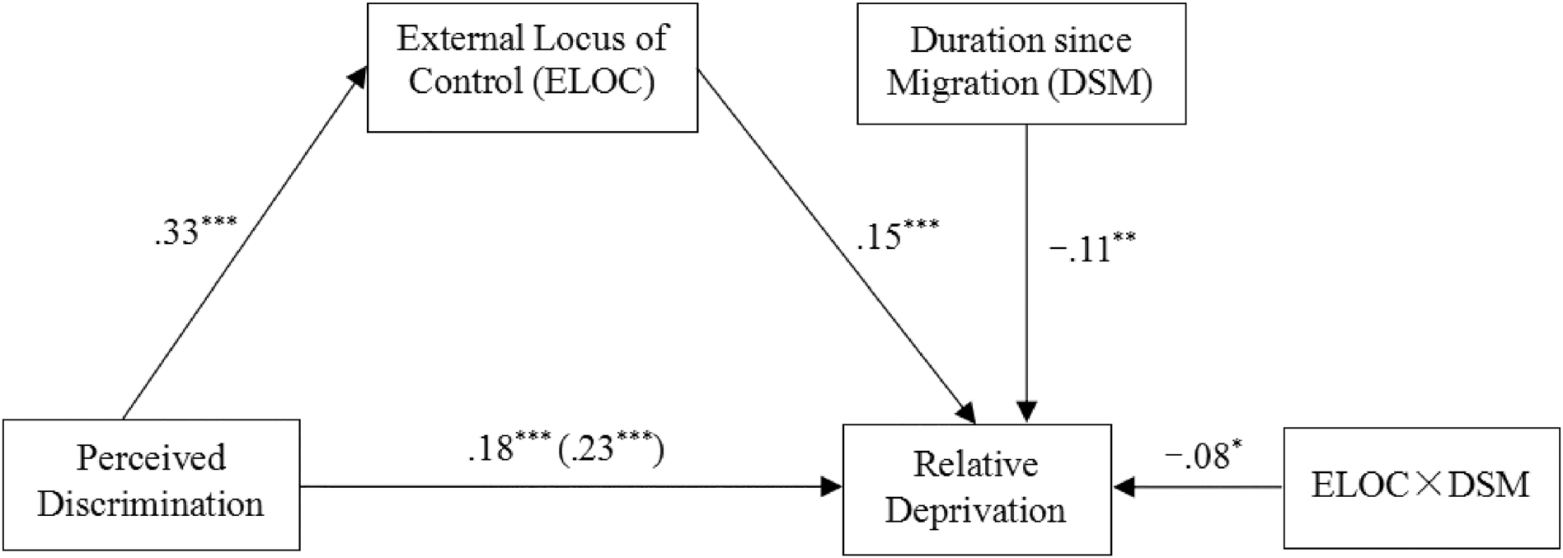
A moderated mediation model with the important results of the paths

To understand the moderating association more clearly, we conducted a separate simple slope analysis for low (1 SD below the mean) and high (1 SD above the mean) levels of duration since migration, using the original scores [81]. The simple slope tests (see Fig. 2) indicated that the association between external locus of control and relative deprivation was weaker for adolescents with long-term migratory duration (*simple slope* = 0.07, *t* = 1.27, *p* > 0.05) than for those with short-term migratory duration (*simple slope* = 0.23, *t* = 4.24, *p* < 0.001). Additionally, the conditional indirect effect test indicated that the indirect association between perceived discrimination and relative deprivation through locus of control was moderated by duration since migration (see Table 3). For adolescents with relatively short-term migratory duration (1 SD below the mean), perceived discrimination was significantly associated with relative deprivation, through increased external locus of control. However, for adolescents with long-term migratory duration (1 SD above the mean), this indirect association was not significant. This difference implies that with a longer duration since migration, the mediating role of external locus of control in the relationship between perceived discrimination and relative deprivation was weaker. Figure 3 shows the important results of the path coefficients for the moderated mediation model.

## Discussion

This study aimed to examine how (mediating mechanisms) and when or for whom (moderating mechanisms) perceived discrimination influences migrant adolescents’ relative deprivation. The results show that locus of control acted as a mediator and that duration since migration acted as a moderator in the relationship between perceived discrimination and relative deprivation. The mediating effect of locus of control was found to differ according to adolescents’ different migratory durations. The indirect link between perceived discrimination and relative deprivation through external locus of control was stronger for migrant adolescents with a short-term migratory duration. Overall, our findings align with social comparison theory [19, 20] and developmental systems theory [57, 58].

### Association between perceived discrimination and relative deprivation

Consistent with previous studies [11, 15], we observed that migrant adolescents who experienced discrimination in destination cities were more likely to feel relative deprivation. Due to differences in lifestyles, values, and cultural backgrounds, migrant adolescents sometimes encounter (explicit or implicit) social exclusion or discrimination in their urban lives [6–8]. When judging that they were treated unjustly, when compared with their urban or rural counterparts, they might feel anger or dissatisfaction with their current situations, resulting in an increasing sense of relative deprivation [13, 26]. In summary, a greater perception of discrimination strengthened migrant adolescents’ sense of relative deprivation [15]. Conversely, when migrant adolescents receive equal treatment, social support, and acceptance in their urban lives, they may gradually integrate into destination cities, which, in turn, reduces their sense of relative deprivation to some extent [82]. Moreover, prior research confirms the positive and direct association between discrimination and poor or fair self-rated health [83] and finds that perspective-taking can effectively combat discrimination denial [84].

### Perceived discrimination, locus of control, and relative deprivation

In line with theoretical expectations and previous studies [53, 56], we observed that locus of control partially mediated the association between perceived discrimination and relative deprivation among migrant adolescents. This indicated that perceived discrimination not only directly affected migrant adolescents’ relative deprivation, it also indirectly affected relative deprivation through external locus of control. According to the analytic model of “internal cause-external cause [85],” the causes of an outcome can be attributed to the person, external circumstances, or a combination of them [86]. It is generally believed that external circumstances are conditions of change, while internal causes are the basis for change [87]. Thus, perceived discrimination can affect relative deprivation by influencing the locus of control of migrant adolescents. These findings imply that migrant adolescents’ external locus of control increases when the perception of discrimination becomes stronger. In other words, migrant adolescents’ locus of control changes, depending on their perceived discrimination. This result can be explained in two ways. First, as new urban residents from rural regions, migrant adolescents’ experiences diminished their ability to exert influence on the people of authority and the environment [88, 89]. Second, adolescents encountering discrimination in cities might feel that the world is an unfair or unjust place,this, in turn, may increase their external locus of control [14].

The present study also found an association between external locus of control and relative deprivation, which suggests that migrant adolescents with an external locus of control reported higher levels of relative deprivation. According to the theory of locus of control [30], as an implicit mental factor, individuals with an external locus of control tend to attribute their outcomes to fate, luck, opportunity, or other external forces. Specifically, migrant adolescents may adopt negative attitudes, emotions, and behaviors to cope with their disadvantaged situations [72], further deteriorating their poor conditions and strengthening their sense of relative deprivation. In summary, locus of control is an important psychological quality for migrant adolescents’ relative deprivation development.

### Moderating effect of duration since migration

Supporting our hypothesis, we observed that the link between external locus of control and relative deprivation was weaker for adolescents with long-term migratory duration than for those with short-term migratory durations. Previous research [29] indicates that duration since migration is an important protective factor attenuating the influence of external environmental factors (e.g., family SES) on migrant adolescents’ relative deprivation; our observations indicate that internal individual factors (i.e., locus of control) can function similarly.

Lazarus and Folkman [90] propose that coping involves both cognitive and behavioral responses used by individuals attempting to manage internal and/or external stressors perceived to exceed their personal resources. Considering this theory, migrating from familiar rural districts to unfamiliar urban regions can be seen as remarkably stressful,to cope with this stressor, individuals tend to seek social resources and support. However, children with relatively short migratory durations have had their existing social support networks disrupted very recently, and new social support networks in their new residences have not yet been established. As a result, they might feel relatively deprived of resources, compared with their urban and rural counterparts [60]. In contrast, migrant adolescents who have lived in the city longer are more likely to have successfully established social support networks (e.g., teachers and peers) and coping resources; therefore, their locus of control may have shifted toward more internal attributions regarding their lives in the city [64]. In summary, duration since migration indeed appears to have an important protective effect that could weaken the influence of external locus of control on migrant adolescents’ relative deprivation.

However, contrary to our expectation, the direct link between perceived discrimination and relative deprivation was not moderated by duration since migration. It could be argued that time-related effects are related to psychological mechanisms (i.e., locus of control), explaining judgments about relative deprivation, rather than general evaluations of perception of discrimination. In other words, locus of control appeared to be the more proximal variable affecting migrant adolescents’ relative deprivation, while perception of discrimination appeared to be a more distal variable, at least among our study participants. In general, among the many factors that affect individual psychosocial development, environmental factors (e.g., parent–child relationship, peer relationship, academic pressure, and social exclusion) are distal variables, while individual factors (e.g., physiology, personality, emotion, cognition, and execution) are proximal variables [91]. Distal variables often exert influence on individuals’ psychosocial development through proximal factors. In this study, discrimination (or perceived discrimination) is an environmental factor, while an external locus of control is an individual factor. Therefore, duration since migration moderated the effects of the proximal variable (i.e., locus of control) on relative deprivation, rather than the effects of the distal variable (i.e., perceived discrimination) on relative deprivation. Future studies should examine this uncovered moderating effect among larger samples and/or multiple regions.

### Implications and limitations

Our findings have clinical implications for Chinese migrant adolescents who are struggling to adapt to their new surroundings and promote community harmony. First, parents and teachers should guide migrant adolescents to foster the positive attributions of their new urban environment, build coping skills that can increase their internal locus of control, and support them in ways that can improve their status (e.g., encourage their involvement in sports teams, introduce them to more stylish clothes, or highlight their special skills and cultural elements from rural life that city children may not know about). Such positive ways of articulating their emotions, fair treatment, and opportunities for individual growth may reduce the relative deprivation of the adolescent migrant population.

Second, urban communities should work toward a fair and inclusive social atmosphere for all residents and always bear in mind that migrants come in to help build their local economies, through which all residents benefit. Specifically, urban communities should strengthen publicity and report positive typical cases of the migrant population to change the urban residents’ negative attitudes toward them. To address this social concern, Balingue [92] proposes solidarity as a means of countering the effects of social discrimination and stigma.

Third, city governments and administrators should ensure that changes in household registration are straightforward, so that migrant adolescents can enroll in local schools readily at academic levels appropriate to their current education; further, having local household residency means that they have complete access to higher-level schools and social welfare, which promotes their personal development in their city lives. Finally, government departments and relevant managers should listen to and seriously consider the opinions of the migrant population, formulate practical and feasible policies and measures, and supervise their implementation. Specifically, they should lower the threshold for migrant workers to settle in the city, promulgate laws and regulations to ensure that the children of migrant workers can enter urban public schools smoothly, and regularly issue living allowances and social welfare to migrant workers’ families.

Although our study had a sizable sample, appropriate measures, and robust analytical methods, it also had several limitations. First, it had a cross-sectional design, which could not confirm the causal relations we assumed among the core variables. Thus, future studies should adopt quasi-experimental designs to further examine the relationships and mechanisms between perceived discrimination and relative deprivation among migrant adolescents. Second, although the self-report method has been widely used to assess perceived discrimination and relative deprivation, and although “self” is the best source of information about perceptions, future research should collect data using multiple methods (i.e., objective measures) and informants (e.g., parents, teachers, and peers). Third, although many environmental and individual variables may affect the relationship between perceived discrimination and relative deprivation, we only focused on two: locus of control and duration since migration. Therefore, future research should consider more proximal variables (e.g., coping style, belief in a just world, and group identification) to accurately clarify when and how perceived discrimination influences migrant adolescents’ relative deprivation. Fourth, although this study measured cognitive and affective components of relative deprivation, we did not measure behaviors or behavioral intention components. Future research should therefore use more comprehensive indicators (e.g., cognitive, emotional, and behavioral items) to measure relative deprivation more objectively. Finally, we only selected migrant adolescents from Fujian province in southeast China as participants, which may limit the representativeness of the sample. Therefore, the findings of this study should be interpreted with caution. Future studies should expand the sample size and representation of migrant adolescents to further validate our findings.

## Conclusion

This study provides a clearer understanding of the mechanisms between perceived discrimination and relative deprivation among Chinese migrant adolescents, as well as the mediating effect of external locus of control and the moderating effect of duration since migration. Furthermore, this study was the first to examine the formation mechanisms of relative deprivation among Chinese migrant adolescents from two perspectives: perceived discrimination and locus of control. The results of this study align with the social comparison theory and developmental systems theory. These results also have implications for improving the mental health of migrant adolescents and the harmony of communities.

## Data Availability

The datasets used and/or analyzed during the current study are available from the corresponding author on reasonable request.
